# A systematic review of the prevalence, risk factors, and protective factors of posttraumatic stress disorder among paramedics

**DOI:** 10.1002/jts.70086

**Published:** 2026-06-04

**Authors:** Brogan Johnston, Danielle Wynne, Michael Threlfall, Kathryn Gardner, Carolina Campodonico

**Affiliations:** ^1^ School of Psychology and Humanities University of Lancashire Preston UK; ^2^ School of Psychology Manchester Metropolitan University Manchester UK

## Abstract

Paramedics are frequently exposed to trauma and may be at elevated risk of posttraumatic stress disorder (PTSD) and complex PTSD (CPTSD). This review aimed to map PTSD prevalence among paramedics, identify risk and protective factors, and scope existing literature on CPTSD in paramedics. Studies were identified through a systematic database search, appraised using the Appraisal Tool for Cross‐Sectional Studies (AXIS) and the Newcastle‐Ottawa Quality Assessment Scale (NOS; for cohort studies). Data were analyzed through narrative synthesis. A total of 43 studies involving 12,783 paramedics were included. PTSD prevalence ranged from 1.0% to 82.4% in the 29 studies that reported prevalence, with 15 studies reporting rates of 14.6%–40%. Consistent risk factors included low social support, poor sleep, workplace stress, anxiety, and depression. Protective factors included resilience, social support, and a sense of coherence. Most cross‐sectional studies were rated as high quality. The limited longitudinal evidence suggests lower PTSD prevalence or symptom levels than cross‐sectional findings. No studies examined CPTSD. Taken together, the findings indicate that PTSD is common among paramedics, although evidence on CPTSD is absent. Future research should prioritize longitudinal designs and examine CPTSD prevalence in paramedics.

Paramedics are exposed to complex, high‐risk situations, increasing their risk of posttraumatic stress disorder (PTSD; Eaton et al., 2021). PTSD, which is characterized by distress triggered by traumatic events, involves avoidance, reexperiencing, and hyperarousal symptoms that can persist long after exposure (Asalgoo et al., 2015). Paramedics report higher PTSD rates than the general population (Hoell et al., 2023), firefighters, and police (Martínez & Blanch, 2024). Despite this, little research addresses risk or protective factors specific to paramedics.

Trauma exposure does not always result in PTSD, suggesting that protective factors like self‐efficacy, a sense of coherence (SoC), and resilience can mitigate risks (Soravia et al., 2021). Studies on firefighters and police show these mechanisms reduce PTSD symptoms (Anders et al., 2022; Schnell et al., 2020), but paramedic‐specific research is limited (Corthésy‐Blondin et al., 2022). Each first responder role carries distinct risks: Police personnel confront violence and crime, firefighters deal with hazardous environments, and paramedics manage medical crises and suicides (Eikenberry et al., 2023; Henderson et al., 2019; Wiegand et al., 2025). Lumping these groups together can obscure such differences. Despite high exposure, paramedics often receive less research and public attention than other responders, partly due to smaller workforce numbers and public perceptions of their role as health care providers rather than front‐line emergency workers (Espinosa, 2025; Johnston et al., 2024).

Complex PTSD (CPTSD), introduced in the *International Statistical Classification of Diseases and Related Health Problems* (11th ed.; *ICD‐11*; World Health Organization [WHO], 2019), includes core PTSD symptoms plus symptoms of affect dysregulation, negative self‐concept, and relationship disturbances (Brewin et al., 2017). CPTSD Prevalence has been reported in police and firefighters (Brewin et al., 2020; Langtry et al., 2021), but no study has examined paramedics, despite their repeated trauma exposure. This systematic review mapped PTSD prevalence among paramedics, explored risk and protective factors, and scoped available evidence on CPTSD. Our aim is that the findings will inform interventions to better support the workforce.

## METHOD

### Search strategy

A systematic search was conducted in PsycInfo, PsycArticles, CINAHL, and MEDLINE (excluding registries). Databases were selected based on institutional access and advice from a subject librarian. Reference lists were also screened. Keywords for paramedics and PTSD/CPTSD were combined with Boolean operators (Table 1). Broader terms (e.g., “burnout,” “risk factors”) were excluded to maximize relevance. Truncation was applied where appropriate. Results were imported into EndNote, and duplicates were removed. Searches were first run in March 2023 and updated in June 2024 and March 2026 with no restrictions.

**TABLE 1:** Keywords and search strings

String	Keywords
String 1	Paramedic* or EMT or “emergency medical technician*” or “ambulance worker*” or EMS OR “emergency medical service*” OR “emergency medical personnel” OR “ambulance personnel” OR “ambulance attendant”
String 2	PTSD OR “posttraumatic stress disorder” OR “trauma” OR posttraumatic OR “post‐traumatic” OR “stress disorder” OR “traumatic stress disorder” OR “Complex post traumatic stress disorder” OR CPTSD OR “complex trauma” OR “complex PTSD” OR “complex post‐traumatic stress” OR “complex posttraumatic stress” OR “complex posttraumatic stress disorder” OR “complex post traumatic stress disorder” OR “complex post‐traumatic stress disorder”

The study protocol was issued ethical clearance by the University of Lancashire's Science Ethics Review Panel (Unique Reference: Science 0262). This study utilized high‐level data from published articles to form the systematic review. As this research did not involve interactions with human subjects, the data used were publicly available, and the study did not envisage any insoluble ethical issues, it was deemed exempt from full ethics review by the University of Lancashire. This systematic review followed PRISMA guidelines (Page et al., 2021).

### Eligibility criteria

Included studies reported original, peer reviewed research on PTSD or CPTSD prevalence, risk, and/or protective factors in paramedics. Members of the research team who reviewed articles included studies on first responders only if the authors reported paramedic data individually and excluded studies on student paramedics due to differing exposure and training levels. The review was limited to studies conducted in Europe, Australia, Canada, and the United States. These regions were selected as they represent high‐income health care systems with broadly comparable paramedic roles, training pathways, and exposure to occupational stressors. By restricting the review to these settings, we aimed to enhance the comparability and applicability of findings to U.K. practice while reducing heterogeneity associated with wider global variation in emergency medical systems and cultural approaches to mental health. To ensure efficient comparisons could be made between results, we excluded articles published before 2013, when the *Diagnostic and Statistical Manual of Mental Disorders* (5th ed.; *DSM‐5*; American Psychiatric Association [APA], 2013) was published. Therefore, articles included in the review spanned 2013–2026.

### Selection process, data extraction, and quality assessment

Two reviewers screened each title and abstract independently, with disagreements resolved by a third reviewer. Full‐text screening and subsequent updates followed the same process. Data extracted included study details (e.g., sample size, study design, study location, measures used), prevalence, mean PTSD scores (where available), risk and protective factors, and key findings. Article exclusion reasons for full text screening can be found in the Supplementary Table S1. Two reviewers independently assessed methodological quality using one of two validated tools: the Appraisal Tool for Cross‐Sectional Studies (AXIS; Downes et al., 2016) for cross‐sectional and mixed‐methods studies, and the Newcastle–Ottawa Scale (NOS) for cohort studies (Wells et al., 2021). Disagreements were resolved by discussion.

### Analysis of results

A narrative synthesis was conducted following Economic and Social Research Council (ESRC) guidance (Popay et al., 2006). Studies were grouped by design to account for methodological differences (Thiese, 2014), then by PTSD measures, prevalence rates, and risk and protective factors.

## RESULTS

### Study selection and study characteristics

Figure 1 outlines the study selection process. After excluding 14 non‐English studies (12 during screening, two at full text screening), a total of 43 studies met the inclusion criteria, comprising 40 cross‐sectional studies and three longitudinal cohort studies. Sample sizes ranged from 31 to 2,656 paramedics, totalling 12,783 paramedics overall. At first glance, there appeared to be overlap between two studies (Rybojad et al., 2019; Rybojad et al., 2019) however, when assessing the results, we determined that although the overall totals were the same for the sample sizes, the paramedic portions differed, and the prevalence reported also differed. There were 21 studies that also included other first responders (e.g., police, firefighters, rescue workers), though the results for paramedics were reported separately. Mean age (reported in 26 studies) ranged from 26 to 45 years. All 43 studies assessed PTSD, posttraumatic stress symptoms (PTSS), or secondary traumatic stress (STS), with 29 reporting prevalence rates and 21 reporting mean scores. No studies on CPTSD met the inclusion criteria. Further details on study characteristics can be found in Table 2.

**FIGURE 1:**
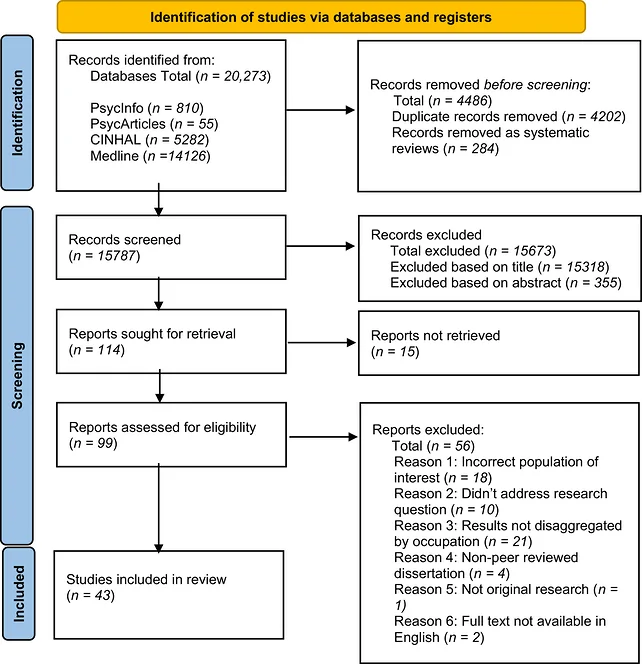
PRISMA flow diagram

**TABLE 2:** Characteristics of the included studies

Article	Sample size & type	Location	PTSD measure	Mean PTSD score	PTSD prevalence	Risk/protective outcomes
*Cross‐sectional: Questionnaire*						
Angehrn et al. (2020)	PSP: *N* = 4,825 (paramedic subsample: *n* = 679	Canada	PCL‐5	22.35	18.4%	Risk: Poor sleep. Insomnia positively correlated with PTSD.
Austin et al. (2018)	Paramedics and EMTs: *N* = 54	United States	STSS			Protective: High resilience. Secondary traumatic stress was negatively correlated with high resilience.
Behnke et al. (2019)	Paramedics: *N* = 102	Germany	PCL‐5 (German version)			Protective: High SoC. Nonsignificantly negatively correlated with PTSD. Less severe PTSD symptoms were significantly negatively associated with the Manageability subscale of the SOC‐13.
Carleton, Afifi, Taillieu, et al(2018)	PSP: *N* = 5,093 (paramedic subsample: *n* = 377)	Canada	PCL‐5			Risk: Chronic pain. Screening positive for PTSD was significantly positively correlated with increased chronic pain.
Carleton, Afifi, Turner, et al. (2018)	PSP: *N* = 5,813 (paramedic subsample: *n* = 311)	Canada	PCL‐5	22.28	24.5%	Risk: Low social support. Social support significantly negatively correlated with PTSD.
D'Alessandro‐Lowe et al. (2024)	PSP: *N* = 314 (paramedic subsample: *n* = 106)	Canada	PCL‐5			Risk: Moral injury was significantly positively associated with PTSD.
Dao‐Tran et al. (2025)	Ambulance personnel: *N* = 492	Australia	PCL‐5		8.4%	Risk: Sexual harassment and maladaptive coping—both were significantly positively correlated with PTSD.
Davis et al. (2019)	Paramedics: *n* = 355, ambulance technicians: *n* = 153	Scotland (United Kingdom)	IES‐R		PTSD symptoms of clinical concern: 14.6% Probable PTSD diagnosis: 10%	
Donnelly & Bennett (2014)	EMS personnel: *N* = 1,633	United States	PCL‐M	29.67		Risk: Critical incident stress—significantly, positively correlated with PTSS.
Donnelly et al. (2016)	Paramedics: *N* = 145	Canada	PCL‐5	30.5		Risk: Critical incident stress, operational stress, and organizational stress; all three significantly positively correlated with PTSS.
Eiche et al. (2019)	Paramedics: *n* = 2,656; emergency physicians: *n* = 75	Germany	PCL‐5 (German version)		5.4% (female: 3.1%, male: 5.9%).	
Frazer et al. (2022)	First responders: *n* = 286 (paramedic subsample: *n* = 61)	Australia	PCL‐5		Paramedics: 16.4%	
Hruska & Barduhn (2021)	EMS personnel: *N* = 79	United States	PCL‐5–Short form			Risk: Poor sleep, high occupational stress, social conflict, and depression severity were significantly negatively correlated with PTSD. Protective: Prosocial impact and recovery activities were significantly positively correlated with PTSD.
Hutchinson et al. (2022)	Ambulance personnel: *N* = 160	United Kingdom	IES‐R	2.11		Risk: Low resilience, perceived stress, and low life satisfaction—resilience and life satisfaction were significantly negatively correlated with PTSD. Perceived stress was significantly positively associated with PTSD.
Khan et al. (2020)	Paramedics: *N* = 194	Australia	GHQ		16%	Risk: Anxiety and depression—PTSD was significantly positively correlated with anxiety and depression
Kobelski et al. (2023)	Health care workers: *N* = 245 (paramedic subsample: *n* = 46)	Poland	PDI	21.24	82.4%	
Köhler et al. (2019)	EMS personnel: *N* = 134	Germany	IES‐R	16.87	16.4%	Risk: Dysfunctional disclosures and negative trauma appraisals were significantly positively correlated with each other; this, in turn, was positively correlated with PTSD.
Kucmin et al. (2018)	Paramedics: *N* = 159	Poland	IES		27.7%	Protective: Emotion‐focused coping and high dispositional optimism were both significantly negatively correlated with PTSD.
Luftman et al. (2017)	First responders: *N* = 546 (EMT subsample: *n* = 318)	United States	PC‐PTSD Screen		Paramedics: 44%	
Maloney et al. (2024)	EMS personnel: *N* = 107 (paramedic subsample: *n* = 68)	United States	PCL‐5		Symptoms: 32.7%, diagnosis: 25.2%	Risk: Low social support—avoiding coworkers was significantly positively associated with PTSD. Protective: Perceived emotional support was significantly negatively associated with PTSD.
Martínez‐Caballero et al. (2021)	First responders: *N* = 317 (EMT subsample: *n* = 178)	Spain	DTS	9.26	30.9%	
Melander et al. (2024)	Ambulance personnel: *N* = 383 (paramedic subsample: *n* = 119)	The Netherlands	ITQ	3.04	Symptoms: 76.8%	Risk: Accessing multiple support types was significantly positively associated with PTSS.
Ogińska‐Bulik et al. (2021)	Paramedics: *n* = 201, nurses: *n* = 218	Poland	STSI	Paramedics: 29.67	Moderate: 56.6%, high: 43.4%	Risk: High workload—workload was significantly positively associated with STS. Protective: High social support and high job satisfaction—both were significantly negatively correlated with STS.
Phillips et al. (2023)	Paramedics: *N* = 90	Australia	NSESSS	5.69		Risk: Ambulance ramping, depression, anxiety, and stress—all were significantly positively correlated with PTSD. Protective: Resilience—significantly negatively correlated with PTSD.
Ragger et al. (2019)	Ambulance personnel: *N* = 266	Austria	IES‐R	5.01		Protective: High SoC—significantly negatively correlated with PTSD.
Reid et al. (2022)	EMTs: *N* = 479	Norway	PTSS‐I‐5		Paramedics: 2.6%, EMTs: 5.9%	Risk: low social support—social support was significantly negatively correlated with PTSD.
Rybojad et al. (2016)	Paramedics: *N* = 100	Poland	IES‐R	1.5	40%	
Rybojad et al. (2019)	EMS personnel: *N* = 100	Poland	IES‐R	22.61	19%	
Setlack et al. (2021)	Paramedics: *n* = 129, firefighters: *n* = 117	Canada	PCL‐5	Paramedics: 20.64	Paramedics: 18.5%	Risk: Workplace violence, depression, and anxiety—all were significantly positively correlated with PTSD.
Shepherd & Wild (2014a)	Ambulance workers: *N* = 45	United Kingdom	PDS‐5	7.4	15.5%	
Shepherd & Wild (2014b)	Ambulance workers: *N* = 45 (paramedic subsample: *n* = 18)	United Kingdom	PDS‐5		Mild: 67%, moderate: 26%, severe: 7%	
Shields et al. (2025)	PSP: *N* = 5,813 (paramedic subsample: *n* = 814)	Canada	PCL‐5		12%	
Sitko‐Dominik et al. (2025)	Paramedics: *N* = 113	Poland	IES‐R	1.46	39.84%	Risk factor: Compliance with antifemininity norms was significantly positively correlated with PTSD.
Skogstad et al. (2015)	PSP: *N* = 238 (ambulance personnel subsample: *n* = 89)	Norway	PCL‐5		Possible PTSD: 1.1% possible PTSD, subthreshold PTSD: 3.4% subthreshold PTSD (ambulance personnel subsample)	
Soravia et al. (2021)	Rescue workers: *N =* 1,002 (ambulance staff subsample: *n =* 97)	Switzerland	PTSS‐I‐5		Ambulance personnel: 15%	Protective: Self‐efficacy—positively associated with PTSS; however, when factoring in the ambulance staff profession, the association was not significant.
Straud et al. (2018)	Paramedic firefighters: *N* = 125	United States	PCL‐C	26.17	Some PTSS endorsement: 73.6%	Risk: Anxiety, depression, and poor sleep—all were significantly positively correlated with PTSD. Protective: Resilience—significantly negatively correlated with PTSD.
Streb et al. (2013)	Paramedics: *N* = 668	Germany (participants were from Switzerland)	PDS‐5		Full PTSD: 4.34%, partial PTSD: 9.58%	Protective: High resilience and high SoC—both were significantly negatively correlated with PTSD.
Tatebe et al. (2020)	First responders: *N* = 258 (paramedic subsample: *n* = 94)	United States	PCL‐5	Paramedics: 17.1	Paramedics: 25%	
Vig et al. (2020)	PSP: *N* = 4,238 (paramedic subsample: *n* = 590)	Canada	PCL‐5			Protective: High social support—social support significantly negatively correlated with PTSD.
Wojciechowska et al. (2023)	Paramedics: *n* = 72, emergency dispatchers: *n* = 66	Poland	IES‐R	54.19		Risk: Alexithymia and ruminations— alexithymia was significantly positively correlated with ruminations, ruminations were significantly positively correlated with PTSD.
*Longitudinal: Cohort*						
Feldman et al. (2021)	EMS personnel: *n* = 135	United States	PCL‐5			Risk: Low social support, poor sleep, depression, and anxiety—at baseline, sleep dysfunction, low social support, depression, and anxiety were significantly positively associated with PTSD; at 3‐month follow‐up, both were still significantly positively associated with PTSD.
Nguyen et al. (2023)	Paramedics: *N =* 101 at baseline, *n =* 93 at follow‐up	Australia	PCL‐5		Baseline: 1%, follow‐up: 2%	
Plaitano et al.) (2025	EMS personnel: *N* = 79 (paramedic subsample: *n* = 31)	United States	PCL‐5–Short Form	2.32		Risk factor: Poor habitual slee—sleep quality was significantly negatively correlated with PTSD.

*Note*: Blank cells indicate data were not reported. PTSD = posttraumatic stress disorder; SoC = sense of coherence; STS = secondary traumatic stress; PCL‐5 = PTSD Checklist for *DSM*‐*5*; PCL‐C = PTSD Checklist–Civilian Version; PCL‐M = PTSD Checklist–Military Version; IES = Impact of Events Scale; IES‐R = Impact of Events Scale–Revised; PDS‐5 = Posttraumatic Stress Diagnostic Scale; PTSS‐I‐5 = Posttraumatic Stress Symptom Scale–Interview for *DSM‐5*; ITQ = International Trauma Questionnaire; GHQ = General Health Questionnaire; DTS = Davidson Trauma Scale; NSESSS = National Stressful Events Survey Acute Stress Disorder—Short Scale; PC‐PTSD‐5 = Primary Care PTSD Screen for *DSM*‐*5*; STISI = Secondary Traumatic Stress Inventory: STSS = Secondary Traumatic Stress Scale.

### PTSD prevalence

A total of 29 studies reported prevalence rates, and 21 reported mean PTSD scores. The most common measures were the PTSD Checklist for *DSM‐5* (PCL‐5; Weathers et al., 2013) or another version of the PCL (21 studies), Impact of Event Scale (IES; Horowitz et al., 1979) or IES‐Revised (IES‐R; Weiss & Marmar, 1997; nine studies), and Posttraumatic Diagnostic Scale for *DSM‐5* (PDS‐5; Foa et al., 2016; three studies), with other tools used sporadically. Prevalence estimates varied widely, with reported rates of 1.0%–73.6% for various iterations of the PCL, 14.6%–40.0% for the IES/IES‐R, and 4.34%–67.0% for the PDS‐5. Outliers, such as a prevalence of 82.4% (Kobelski et al., 2023) reported in one study using the Peritraumatic Distress Inventory (PDI; Brunet et al., 2001), reflected methodological and out‐of‐population differences. Despite this heterogeneity, most prevalence estimates (15 out of 29) fell between 14.6% and 40.0%.

Two of the three longitudinal studies used the full PCL‐5, and the third used a short‐form version of the measure. The study that used the shortened measure reported a mean PTSD score of 2.32 (Plaitano et al., 2025), another study found that prevalence increased slightly from 1.0% to 2.0% from baseline to follow‐up in newly qualified paramedics (Nguyen et al., 2023), and the third study focused on predictors rather than prevalence (Feldman et al., 2021). Collectively, the longitudinal studies suggested lower PTSD prevalence than cross‐sectional studies, though their methodological quality was limited. See Table 2 for details about individual studies.

### Risk factors

A total of 22 studies identified potential PTSD risk factors. The most consistently reported risk factors were low social support, workplace stress, poor sleep, and preexisting anxiety or depression. Low social support was linked to higher PTSD symptom severity across five studies, including deficits in family, peer, and supervisor support. Five studies found that work‐related stress was significantly associated with PTSD, encompassing chronic workplace stress, operational stress, and critical incident stress. Poor sleep was associated with PTSD in five studies, typically involving reduced sleep quality, sleep duration, or insomnia. Preexisting symptoms of anxiety and depression were linked to PTSD in six studies. One study also identified alexithymia as a potential risk factor, particularly when associated with rumination, and one study identified low resilience as a potential risk factor. Table 2 details risk factors found in each study.

### Protective factors

Twelve studies examined protective factors. Resilience was the most frequently reported protective factor, with four studies identifying a negative association with PTSD severity; in some cases, resilience mediated the impact of depressive symptoms or was linked with life satisfaction to ultimately impact PTSD. Three studies assessed SoC, demonstrating significant inverse associations with PTSD, with stronger scores linked to reduced symptom severity. Finally, four studies reported that high social or emotional support, particularly from family and peers, was protective against PTSD. Taken together, these findings indicate that resilience, SoC, and social support operate as important buffers against PTSD in paramedics. Table 2 details protective factors found in each study.

### Risk of bias

Because the AXIS lacks formal scoring systems, the review followed approaches used in previous research (Sahu et al., 2020), assigning 1 point for each criterion met. We considered AXIS scores of 14–20 to be high quality/low risk, 7–13 to be medium quality/medium risk, and 0–6 to be low quality/high risk. Of the 40 cross‐sectional questionnaire studies, 27 were rated as high quality and 13 as medium quality, with none classified as low quality (Figure 3). For the NOS, which was applied to longitudinal cohort studies, we used established classifications (Wells et al., 2021). Of the three longitudinal cohort studies, one was categorized as low quality, one as medium quality, and one as high quality (Figure 2). The most common issues were inadequate justification of sample sizes; limited information on funding, conflicts of interest, and ethics; and potential self‐selection or nonresponse bias. Detailed quality scores for individual studies are provided in the Supplementary Table S2.[Fig jts70086-fig-0003]

**FIGURE 2:**
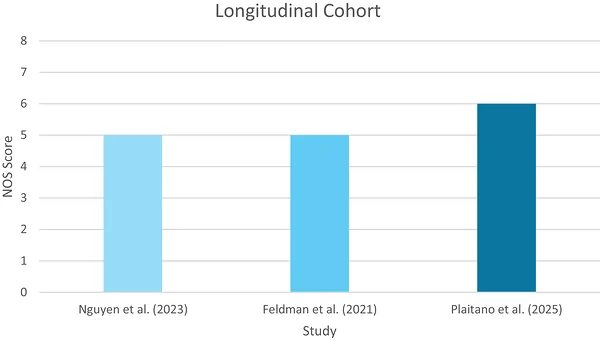
Risk of bias and quality assessment for longitudinal cohort studies *Note*: NOS = Newcastle–Ottawa Quality Assessment Scale

**FIGURE 3 jts70086-fig-0003:**
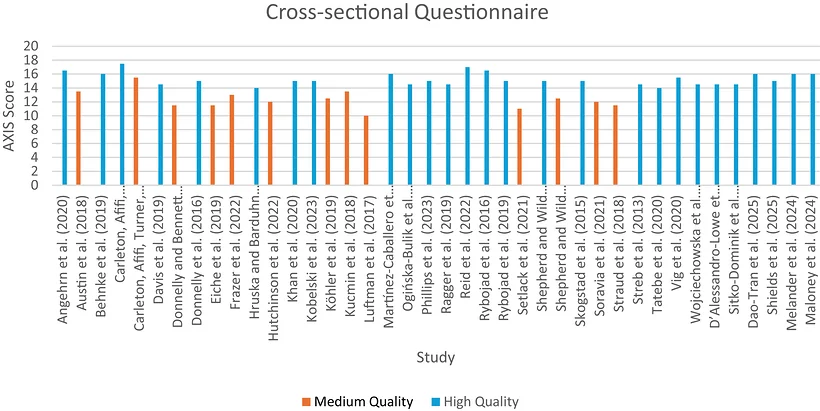
Risk of bias and quality assessment for cross‐sectional questionnaire studies *Note*: AXIS = Appraisal Tool for Cross‐Sectional Studies.

## DISCUSSION

This systematic review examined PTSD prevalence among paramedics, explored associated risk and protective factors, and explored the extent to which CPTSD has been investigated within this population. The findings indicate that PTSD is a significant concern among paramedics, with prevalence estimates varying widely but consistently clustering within a moderate range. The absence of research on CPTSD highlights a critical gap in the literature, particularly given the repeated and cumulative nature of trauma exposure in this occupational group.

A first key finding concerns the substantial variability in reported PTSD prevalence, with estimates ranging from 1.0% (Nguyen et al., 2023) to 82.4% (Kobelski et al., 2023), although most studies reported prevalence within a narrower band, typically between 14.6% and 40%. This pattern suggests methodological differences, with studies employing the PCL‐5, aligned with *DSM‐5*, generally reporting lower prevalence than those using older or less‐specific instruments, such as the IES‐R. These discrepancies mirror wider evidence showing that prevalence rates differ when *DSM* and *ICD* criteria are applied, with *ICD‐11–*based measures often producing lower estimates (Barbano et al., 2018; Wisco et al., 2016). Differences in publication period may also contribute, given the transition from the *DSM*‐*IV* (APA, 1994) to the *DSM‐5*; to minimize this, only studies published after the publication of the *DSM‐5* were included. Geographical differences further contributed to variability, with higher PTSD rates observed in the United States compared to the United Kingdom. This may reflect differences in training, professional structure, and autonomy. U.K. paramedics typically receive more formalized training and operate within the National Health Service (NHS), whereas U.S. systems are more fragmented, often with shorter training pathways and varying levels of autonomy (Feerick et al., 2025). Broader cross‐national research suggests that both trauma exposure and cultural attitudes toward mental health influence outcomes (Benjet et al., 2016; Krendl & Pescosolido, 2020). At the same time, the underreporting of PTSD symptoms remains a possibility across all contexts due to stigma, particularly self‐stigma, which may persist even when public stigma declines (Lewis et al., 2022). Despite this heterogeneity, most studies reported a prevalence between 14.6% and 40%, substantially higher than general population estimates (Hoell et al., 2023), confirming that PTSD represents a significant occupational health concern among paramedics.

A second key finding relates to the identification of consistent risk factors. Across studies, several factors were consistently associated with higher PTSD scores in paramedics. Low social support was repeatedly identified as a risk factor, with deficits in family, peer, and/or supervisor support linked to higher levels of PTSD symptom severity (Carleton, Afifi, Turner, et al., 2018; Feldman et al., 2021; Maloney et al., 2024; Reid et al., 2022). Although this reflects the established buffering role of social support (Cohen & Wills, 1985), it also highlights the particular vulnerability of paramedics, who often work in isolation and may lack immediate peer support compared to other first responders (Cogan et al., 2025). Workplace stress was another prominent risk factor, with chronic organizational pressures, operational demands, and exposure to critical incidents (Donnelly et al., 2016) associated with higher PTSD scores. Evidence suggests that PTSD symptom severity increases with cumulative occupational stressors (Hruska & Barduhn, 2021) and further supports the importance of considering ongoing stress exposure rather than isolated events.

Sleep disturbance also emerged as an important correlate. Insomnia, reduced sleep duration, and poor sleep quality were linked to higher PTSD risk (Angehrn et al., 2020; Feldman et al., 2021; Straud et al., 2018). Sleep disruption may both result from trauma exposure (Straud et al., 2018) and contribute to symptom maintenance through mechanisms such as heightened arousal (Han et al., 2012) and cortisol dysregulation (Gerding & Wang, 2022). In paramedic settings, irregular shifts and extended working hours may further exacerbate these effects. There was evidence of strong associations between preexisting mental health symptoms and PTSD, with higher levels of anxiety and depression predicting higher PTSD prevalence (Phillips et al., 2023). However, the direction of this association remains unclear, as these conditions may increase vulnerability to PTSD (Isaac et al., 2024), whereas PTSD may also exacerbate mood and anxiety symptoms (Ma et al., 2025), suggesting a reciprocal relationship. Additional factors were less frequently examined but may be relevant. For example, alexithymia, particularly in combination with rumination, was identified as a potential risk factor (Wojciechowska et al., 2023), suggesting that difficulties in emotional processing may hinder trauma recovery.

Protective factors were less extensively examined but showed consistent patterns. Resilience was the most frequently identified protective factor and was associated with lower PTSD symptom severity (Hutchinson et al., 2022) and, in some cases, mediated the association between depression and PTSD (Straud et al., 2018). A stronger SoC was also linked to reduced symptom severity (Streb et al., 2014). These factors may operate synergistically, as resilience and SoC together explained a substantial proportion of variance in PTSD severity, consistent with Hobfoll's (1989) conservation of resources theory, which proposes that psychological and social resources cluster and reinforce one another. Taken together, these findings suggest that PTSD in paramedics is shaped by a complex interplay of occupational, social, and personal factors, and that interventions targeting multiple domains may be most effective.

A notable finding of this review is the absence of studies examining CPTSD in paramedic populations. This is particularly striking given that CPTSD is conceptualized as arising from prolonged or repeated trauma exposure (Brewin et al., 2017), which may be characteristic of paramedic work. Evidence from related populations suggests that CPTSD may be relevant in occupational contexts involving repeated trauma (Brewin et al., 2020; Langtry et al., 2021; Øvstebø et al., 2023), yet this has not been empirically examined in paramedics. This gap limits understanding of whether existing PTSD frameworks fully capture the complexity of trauma responses in this group or whether a subset of paramedics may experience more pervasive disturbances consistent with CPTSD.

These findings have several implications for research and practice. There is a clear need for longitudinal studies to examine the development and course of PTSD symptoms over time, as well as the potential emergence of CPTSD. Such work would allow for a more nuanced understanding of how repeated trauma exposure interacts with individual and organizational factors. From a practical perspective, the identification of modifiable risk factors, including workplace stress and sleep disruption, highlights potential targets for intervention. Organizational strategies aimed at improving working conditions, promoting supportive environments, and facilitating access to psychological support may, therefore, play an important role in reducing PTSD risk.

Strengths of this review include a comprehensive multidatabase search strategy, developed with librarian input, supplemented by reference list checks and an updated search to maximize coverage. Screening, data extraction, and quality appraisal were conducted independently by multiple reviewers, enhancing rigor and minimizing bias. The protocol was preregistered, and PRISMA guidelines were followed, further strengthening transparency. In terms of limitations, we acknowledge that the predominance of cross‐sectional studies limits causal inference (Wang & Cheng, 2020), and the limited number of longitudinal studies restricts insight into symptom trajectories over time. The use of AXIS, while appropriate, involves some subjectivity due to the absence of formal scoring, despite mitigation through established scoring brackets (Sahu et al., 2020). Inconsistent reporting across studies also complicated synthesis, with variation in the reporting of prevalence, mean scores, and methodological details. Generalizability may be limited, as studies were restricted to high‐income Western health care systems, and sample representativeness may be affected by the predominance of male participants (Health & Care Professions Council, 2023), potentially limiting insights into sex and/or gender differences (Özbay & Bülbül, 2025). Finally, volunteer bias may have influenced findings, as individuals with differing levels of distress may be more or less likely to participate (Cheung et al., 2017).

In conclusion, PTSD appears to be a prevalent concern among paramedics and is shaped by a combination of individual, interpersonal, and organizational factors. However, the current evidence base remains limited by a lack of longitudinal research and a complete absence of studies examining CPTSD. Addressing these gaps will be essential for developing a more comprehensive understanding of trauma‐related difficulties in this population, as well as for informing effective support and intervention strategies.

## AUTHOR NOTE

This research did not receive any specific grant from funding agencies in the public, commercial, or not‐for‐profit sectors.

## OPEN PRACTICES STATEMENT

The study was preregistered with PROSPERO on August 29, 2023 (CRD42023458123). The summary data that support the findings of this study are available in the supplementary material of this article. The materials used in these studies are widely publicly available. Neither the data nor the materials have been made available on a permanent third‐party archive

## AUTHOR CONTRIBUTIONS

**Brogan Johnston**: conceptualization, methodology, investigation, data curation, writing—original draft, writing—review and editing, visualization, project administration. **Carol Campodonico**: conceptualization, writing—review and editing, supervision. **Danielle Wynne**: data curation, writing—review and editing. **Michael Threlfall**: data curation, writing—review and editing. **Kathryn Gardner**: conceptualization, writing—review and editing, supervision.

## Supporting information



SUPPORTING INFORMATION
