# Supplementary material for: A systematic review of the prevalence, risk factors, and protective factors of posttraumatic stress disorder among paramedics
Source: J Trauma Stress. 2026 Jun 4;39(5):745–60. doi: 10.1002/jts.70086 (PMC13639195; doi:10.1002/jts.70086)
Supplement: Supplementary file 1 [file JTS-39-745-s001.docx]

**Supplementary Table S1: Articles Excluded During Full Text Screening**

| Author | Title | Reason for Exclusion |
| --- | --- | --- |
| Haskins et al. (2021) | A binational survey of smartphone activated volunteer responders for out-of-hospital cardiac arrest: Availability, interventions, and post-traumatic stress | Didn’t report paramedic PTSD prevalence/protective factors/risk factors |
| Groll et al. (2019) | A cross-sectional study of the relationship between previous military experience and mental health disorders in currently serving public safety personnel in Canada | Results were not broken down by occupation |
| Wild et al. (2016) | A prospective study of pre-trauma risk factors for post-traumatic stress disorder and depression | Student paramedics only - Incorrect population |
| Lubin et al. (2007) | Acute stress disorder and post-traumatic stress disorder among medical personnel in Judea and Samaria areas in the years 2000-2003 | Did not include paramedics – Incorrect population |
| Jonker et al. (2020) | An intervention framework to facilitate psychological trauma management in high-risk occupations | Didn’t report paramedic PTSD prevalence/protective factors/risk factors |
| Nagamine et al. (2020) | Assessment of Factors Associated With Long-term Posttraumatic Stress Symptoms Among 56 388 First Responders After the 2011 Great East Japan Earthquake | Not peer reviewed |
| Meckes et al. (2021) | Association between physical activity and mental health among first responders with different service roles | Did not include paramedics - Incorrect population |
| Halpern et al. (2011) | Attachment insecurity, responses to critical incident distress, and current emotional symptoms in ambulance workers | Didn’t report paramedic PTSD prevalence/protective factors/risk factors |
| Soffer et al. (2011) | Correlations between psychosocial factors and psychological trauma symptoms among rescue personnel | Didn’t report paramedic PTSD prevalence/protective factors/risk factors |
| Ward (2006) | Critical incident exposure in South African emergency services personnel: prevalence and associated mental health issues | Results were not broken down by occupation |
| Bromet et al. (2015) | DSM-IV post-traumatic stress disorder among World Trade Center responders 11–13 years after the disaster of 11 September 2001 (9/11) | Results were not broken down by occupation |
| Greenblatt‐Kimron et al. (2022) | Early maladaptive schemas and ICD-11 CPTSD symptoms: Treatment considerations | Did not include paramedics - Incorrect population |
| Dearing and Kippenbrock (2022) | Emergency Transport Crew: Post-Traumatic Stress Disorder Prevention Program | Not peer reviewed |
| Mann et al. (2021) | Exploring resilience in undergraduate and early career paramedics | Student paramedics only - Incorrect population |
| Carleton et al. (2019) | Exposures to Potentially Traumatic Events Among Public Safety Personnel in Canada | Didn’t report paramedic PTSD prevalence/protective factors/risk factors |
| Motreff et al. (2020) | Factors associated with PTSD and partial PTSD among first responders following the Paris terror attacks in November 2015 | Did not include paramedics - Incorrect population |
| Declercq et al. (2011) | Frequency of and subjective response to critical incidents in the prediction of PTSD in emergency personnel | Did not include paramedics - Incorrect population |
| Jasielska and Ziarko (2019) | General and specific individual post-traumatic stress disorder-related mechanisms among paramedics | Not published in English |
| Behnke et al. (2019) | Higher sense of coherence is associated with better mental and physical health in emergency medical services: Results from investigations on the Revised Sense of Coherence Scale (SOC-R) in rescue workers | Doesn’t differentiate types of rescue workers - Incorrect population |
| Wild and Chang (2022) | Is It Personal? The Effect of Personal vs. Occupational Trauma on PTSD Symptom Severity in Emergency Responders | Results were not broken down by occupation |
| Aldrich and Cerel (2020) | Occupational Suicide Exposure and Impact on Mental Health: Examining Differences Across Helping Professions | Did not include paramedics - Incorrect population |
| Jones et al. (2018) | Prevalence and correlates of psychiatric symptoms among first responders in a Southern State | Results were not broken down by occupation |
| Kang et al. (2015) | Psychological consequences and quality of life among medical rescuers who responded to the 2010 Yushu earthquake: A neglected problem | Did not include paramedics - Incorrect population |
| Zhao et al. (2019) | Self-Acceptance, Post-Traumatic Stress Disorder, Post-Traumatic Growth, and the Role of Social Support in Chinese Rescue Workers | Did not include paramedics - Incorrect population |
| Van Der Velden et al. (2008) | Smoking predicts posttraumatic stress symptoms among rescue workers: A prospective study of ambulance personnel involved in the Enschede fireworks disaster | Didn’t report paramedic PTSD prevalence/protective factors/risk factors |
| Ogińska-Bulik (2015) | Social support and negative and positive outcomes of experienced traumatic events in a group of male emergency service workers | Did not include paramedics - Incorrect population |
| Kshtriya et al. (2020) | Social support as a mediator of occupational stressors and mental health outcomes in first responders | Results were not broken down by occupation |
| Øvstebø et al. (2023) | Symptoms of post-traumatic stress disorder among first aid providers | Did not include paramedics – Incorrect population |
| Halpern et al. (2012) | The critical incident inventory: characteristics of incidents which affect emergency medical technicians and paramedics | Didn’t report paramedic PTSD prevalence/protective factors/risk factors |
| Noda et al. (2022) | The mediating effect of symptoms of posttraumatic stress disorder and depression on the relationship between personality traits and quality of life in emergency service workers | Results were not broken down by occupation |
| Kılıç and İnci (2015) | Traumatic Stress in Emergency Medical Technicians: Protective Role of Age and Education | Didn’t report paramedic PTSD prevalence/protective factors/risk factors |
| Boehme et al. (2023) | Factor structure and factorial invariance of the PTSD checklist for DSM-5 in public safety personnel: Results from a large and diverse sample | Didn’t report paramedic PTSD prevalence/protective factors/risk factors |
| Reffi and Cheng (2023) | Healthy sleep may bolster resilience to depression and posttraumatic stress disorder in new paramedics | Not original research, review of other research |
| Gurowiec et al. (2023) | The mediating role of job satisfaction in the relationship between social support and the consequences of secondary exposure to trauma among medical providers working with trauma victims | Results were not broken down by occupation |
| Beattie et al. (2023) | Network analysis of posttraumatic stress disorder in a treatment-seeking sample of US firefighters and emergency medical technicians | Didn’t report paramedic PTSD prevalence/protective factors/risk factors |
| Turman (2019) | No appointment necessary: Emotion regulation and posttraumatic stress disorder in emergency medical technicians and paramedics | Not peer reviewed |
| Alexandrov et al. (2024) | Posttraumatic stress disorder symptomology as measured by PCL-5 and its relationships to resilience, hostility and stress among paramedics and social professionals | Results were not broken down by occupation |
| Roig (2021) | A quantitative study exploring dynamics of perceived secondary trauma in first responders | Not peer reviewed |
| Alexandrov et al. (2024) | Resilience in Emergency Medicine during COVID-19: Evaluating Staff Expectations and Preparedness | Results were not broken down by occupation |
| Ogińska-Bulik (2023) | The role of cognitive trauma processing in the occurrence of secondary traumatic stress symptoms in professionals working with people after traumatic experiences | Not published in English |
| Kaufman et al. (2024) | Spirituality/Religion Among First Responders With and Without Posttraumatic Stress Disorder and Alcohol Misuse | Results were not broken down by occupation |
| Kinany et al. (2025) | Impact of patient death on physicians’ mental health | Full text did not refer to paramedics – Incorrect population |
| Ahmad et al. (2025) | Network analysis of secondary traumatic stress, coping, and post-traumatic growth in frontline healthcare workers during COVID-19 outbreak | Results were not broken down by occupation |
| Kaufman et al. (2024) | Spirituality/Religion Among First Responders With and Without Posttraumatic Stress Disorder and Alcohol Misuse | Results were not broken down by occupation |
| Laing et al. (2026) | Supporting Healthcare and Paramedic Employees (SHAPE)-study protocol for a single-blind, superiority randomised controlled trial evaluating cognitive therapy coaching for PTSD and MDD for health and social care workers in the United Kingdom | Full text did not refer to paramedics – Incorrect population |
| Alexandrov et al. (2024) | Posttraumatic stress disorder symptomology as measured by PCL‐5 and its relationships to resilience, hostility and stress among paramedics and social professionals | Results were not broken down by occupation |
| Harenberg et al. (2025) | Team Cohesion and Mental Disorder Symptoms in Canadian Helicopter Emergency Medical Service Personnel | Full text did not refer to paramedics – incorrect population |
| Kaufman et al. (2024) | The association between PTSD symptom clusters and religion/spirituality with alcohol use among first responders | Results were not broken down by occupation |
| Çelebi et al. (2025) | The impact of a huge earthquakes on the mental health of first responders | Results were not broken down by occupation |
| Goniewicz et al. (2024) | The Silent Burden: Investigating Post-Traumatic Stress Disorder and Social Isolation Among Healthcare Workers During COVID-19 | Results were not broken down by occupation |
| Bryant et al. (2025) | Augmenting trauma-focused cognitive behaviour therapy for post-traumatic stress disorder with memory specificity training: a randomized controlled trial | Results were not broken down by occupation |
| Woller et al. (2025) | Examining moral injury and posttraumatic stress among firefighters, emergency medical services personnel, and emergency dispatchers | Results were not broken down by occupation |
| Wild et al. (2026) | Cognitive Resilience Training to Prevent PTSD and Major Depressive Disorder  in Paramedic Recruits | Student paramedics only - Incorrect population |
| Włoszczak-Szubzda et al. (2025) | Predictors of post-traumatic stress disorder among healthcare workers during the COVID-19 pandemic in Poland | Full text did not refer to paramedics – incorrect population |
| Carleton et al. (2025) | Mental health disorder symptom changes among public safety personnel  after emotional resilience skills training | Results were not broken down by occupation (only mean scores were) |
| Sopp et al. (2025) | How We See the World: Inflexible Interpretation Updating as a Predictor  and Moderator of PTSD Symptoms in High-Risk Occupations | Results were not broken down by occupation |

**Supplementary Table S2: Quality Appraisal Results**

| Reference | Study Design | Critical Appraisal Tool | Critical Appraisal Checklist Score | Quality Rating |
| --- | --- | --- | --- | --- |
| Angehrn et al. (2020) | Cross-sectional- Questionnaire | Appraisal for cross-sectional studies AXIS | 16.5 out of 20 | High |
| Austin et al. (2018) | Cross-sectional- Questionnaire | Appraisal for cross-sectional studies AXIS | 13.5 out of 20 | Medium |
| Behnke et al. (2019) | Cross-sectional- Questionnaire | Appraisal for cross-sectional studies AXIS | 16 out of 20 | High |
| Carleton et al. (2018) | Cross-sectional- Questionnaire | Appraisal for cross-sectional studies AXIS | 17.5 out of 20 | High |
| Carleton et al. (2017) | Cross-sectional- Questionnaire | Appraisal for cross-sectional studies AXIS | 15.5 out of 20 | Medium |
| Davis et al. (2019) | Cross-sectional- Questionnaire | Appraisal for cross-sectional studies AXIS | 14.5 out of 20 | High |
| Donnelly and Bennett (2014) | Cross-sectional- Questionnaire | Appraisal for cross-sectional studies AXIS | 11.5 out of 20 | Medium |
| Donnelly et al. (2015) | Cross-sectional- Questionnaire | Appraisal for cross-sectional studies AXIS | 15 out of 20 | High |
| Eiche et al. (2019) | Cross-sectional- Questionnaire | Appraisal for cross-sectional studies AXIS | 11.5 out of 20 | Medium |
| Frazer et al. (2022) | Cross-sectional- Questionnaire | Appraisal for cross-sectional studies AXIS | 13 out of 20 | Medium |
| Hruska and Barduhn (2021) | Cross-sectional- Questionnaire | Appraisal for cross-sectional studies AXIS | 14 out of 20 | High |
| Hutchinson et al. (2022) | Cross-sectional- Questionnaire | Appraisal for cross-sectional studies AXIS | 12 out of 20 | Medium |
| Khan et al. (2020) | Cross-sectional- Questionnaire | Appraisal for cross-sectional studies AXIS | 15 out of 20 | High |
| Kobelski et al. (2023) | Cross-sectional- Questionnaire | Appraisal for cross-sectional studies AXIS | 15 out of 20 | High |
| Köhler et al. (2019) | Cross-sectional- Questionnaire | Appraisal for cross-sectional studies AXIS | 12.5 out of 20 | Medium |
| Kucmin et al. (2018) | Cross-sectional- Questionnaire | Appraisal for cross-sectional studies AXIS | 13.5 out of 20 | Medium |
| Luftman et al. (2017) | Cross-sectional- Questionnaire | Appraisal for cross-sectional studies AXIS | 10 out of 20 | Medium |
| Martínez-Caballero et al. (2021) | Cross-sectional- Questionnaire | Appraisal for cross-sectional studies AXIS | 16 out of 20 | High |
| Ogińska-Bulik et al. (2021) | Cross-sectional- Questionnaire | Appraisal for cross-sectional studies AXIS | 14.5 out of 20 | High |
| Phillips et al. (2022) | Cross-sectional- Questionnaire | Appraisal for cross-sectional studies AXIS | 15 out of 20 | High |
| Ragger et al. (2019) | Cross-sectional- Questionnaire | Appraisal for cross-sectional studies AXIS | 14.5 out of 20 | High |
| Reid et al. (2022) | Cross-sectional- Questionnaire | Appraisal for cross-sectional studies AXIS | 17 out of 20 | High |
| Rybojad et al. (2016) | Cross-sectional- Questionnaire | Appraisal for cross-sectional studies AXIS | 16.5 out of 20 | High |
| Rybojad et al. (2019) | Cross-sectional- Questionnaire | Appraisal for cross-sectional studies AXIS | 15 out of 20 | High |
| Setlack et al. (2021) | Cross-sectional- Questionnaire | Appraisal for cross-sectional studies AXIS | 11 out of 20 | Medium |
| Shepherd and Wild (2013) | Cross-sectional- Questionnaire | Appraisal for cross-sectional studies AXIS | 15 out of 20 | High |
| Shepherd and Wild (2014) | Cross-sectional- Questionnaire | Appraisal for cross-sectional studies AXIS | 12.5 out of 20 | Medium |
| Skogstad et al. (2015) | Cross-sectional- Questionnaire | Appraisal for cross-sectional studies AXIS | 15 out of 20 | High |
| Soravia et al. (2021) | Cross-sectional- Questionnaire | Appraisal for cross-sectional studies AXIS | 12 out of 20 | Medium |
| Straud et al. (2018) | Cross-sectional- Questionnaire | Appraisal for cross-sectional studies AXIS | 11.5 out of 20 | Medium |
| Streb et al. (2013) | Cross-sectional- Questionnaire | Appraisal for cross-sectional studies AXIS | 14.5 out of 20 | High |
| Tatebe et al. (2020) | Cross-sectional- Questionnaire | Appraisal for cross-sectional studies AXIS | 14 out of 20 | High |
| Vig et al. (2020) | Cross-sectional- Questionnaire | Appraisal for cross-sectional studies AXIS | 15.5 out of 20 | High |
| Wojciechowska et al. (2023) | Cross-sectional- Questionnaire | Appraisal for cross-sectional studies AXIS | 14.5 out of 20 | High |
| D’Alessandro‐Lowe et al. (2024) | Cross-sectional- Questionnaire | Appraisal for cross-sectional studies AXIS | 14.5 out of 20 | High |
| Sitko-Dominik et al. (2025) | Cross-sectional- Questionnaire | Appraisal for cross-sectional studies AXIS | 14.5 out of 20 | High |
| Dao‐Tran et al. (2025) | Cross-sectional- Questionnaire | Appraisal for cross-sectional studies AXIS | 16 out of 20 | High |
| Shields et al. (2025) | Cross-sectional- Questionnaire | Appraisal for cross-sectional studies AXIS | 15 out of 20 | High |
| Melander et al. (2024) | Cross-sectional- Questionnaire | Appraisal for cross-sectional studies AXIS | 16 out of 20 | High |
| Maloney et al. (2024) | Cross-sectional- Questionnaire | Appraisal for cross-sectional studies AXIS | 16 out of 20 | High |
| Nguyen et al. (2023) | Longitudinal- Cohort | Newcastle- Ottawa Quality Assessment Scale  Cohort Studies | 5 out of 8 | Fair/Medium |
| Feldman et al. (2021) | Longitudinal- Cohort | Newcastle- Ottawa Quality Assessment Scale  Cohort Studies | 5 out of 8 | Poor/Low |
| Plaitano et al. (2025) | Longitudinal- Cohort | Newcastle- Ottawa Quality Assessment Scale  Cohort Studies | 6 out of 8 | Good/High |
